# The Distribution of *Blastocystis* Subtypes among School-aged Children in Mugla, Turkey

**Published:** 2017

**Authors:** Funda SANKUR, Seniz AYTURAN, Erdogan MALATYALI, Hatice ERTABAKLAR, Sema ERTUG

**Affiliations:** 1. Microbiology Laboratory, Training and Research Hospital, Mugla Sitki Kocman University, Mugla, Turkey; 2. Dept. of Parasitology, Faculty of Medicine, Adnan Menderes University, Aydin, Turkey

**Keywords:** *Blastocystis*, Subtype, Children, Turkey

## Abstract

**Background:**

*Blastocystis* is a common protozoon that inhabits human intestinal tract and has a worldwide distribution. This study aimed to determine subtype (ST) distribution of *Blastocystis* among school-aged children in a western city of Turkey between Mar and Jun 2014.

**Methods:**

This cross-sectional study was conducted among primary school children in Mugla between Mar and Jun 2014. Overall, 468 stool samples from children were examined by direct microscopy and inoculated into Jones medium. *Blastocystis* partial small subunit ribosomal RNA gene (SSU-rDNA) was amplified and sequenced from culture positive isolates. Subtypes were determined according to closest or exact match at GenBank and *Blastocystis* ST (18S) database.

**Results:**

The positive rate of *Blastocystis* was 7.4% (n=35) with xenic in-vitro culture (XIVC). The subtypes could be identified for 33 (94.2%) isolates; 12 (34.2%) were ST3, 11 (31.4%) were ST1, 9 (25.7%) were ST2, one was (2.8%) ST7. No relationship was found between *Blastocystis* infected and non-infected cases in terms of gastrointestinal symptoms. Additionally, none of the possible risk factors was related to *Blastocystis* infection.

**Conclusion:**

Subtypes in children was similar to those reported in most of the studies that found ST3 as the most common subtype.

## Introduction

*Blastocystis* is a common enteric protozoon with a worldwide distribution. High infection rates of *Blastocystis* have been reported in developing/undeveloped countries as compared to industrialized ones (1). Additionally, similar to other intestinal parasitic infections, children in underdeveloped countries have more susceptibility to *Blastocystis* infection because of poverty-related factors (2).

Recently, researchers have shown an increased interest in pathogenesis of *Blastocystis*; however, offers contradictory findings of the pathogenesis of *Blastocystis* (3). *Blastocystis* has a long-term host colonization and might be common and diverse member of healthy intestinal flora (4). A link was revealed between *Blastocystis* and diseases such as irritable bowel syndrome and urticaria (5, 6). Moreover, *Blastocystis* may be an opportunistic pathogen in immune-compromised patients (7). Microscopy, xenic in-vitro culture (XIVC), and molecular biology-based techniques are used commonly used for the detection of *Blastocystis* diagnosis in routine parasitology laboratories and epidemiological studies (8). Molecular methods have gained importance in recent years as they have highest sensitivities and allow identifying subtypes (STs) (9). Despite the increased information on subtype distribution of *Blastocystis*, there is still lack of data from many parts of world.

To date, 17 subtypes of *Blastocystis* have been identified from different hosts and nine of them are known to cause infection in humans (1). The most diverse *Blastocystis* subtypes in humans all around the world are ST1-ST4 which accounts for 90% of total isolates (10). Currently, there are three main approaches as the method of choice for *Blastocystis* subtyping sequencing of small subunit rRNA gene (SSU-rDNA) PCR products called as “barcoding”, PCR with subtype-specific sequence-tagged-site (STS) primers STS-PCR and real-time PCR (9, 11).

Several studies deal with *Blastocystis* subtypes in Turkey, ST1, 2,3,4,6 and 7 was isolated from humans in different populations in Turkey (12, 13). However, to date, no previous study has investigated the ST distribution of *Blastocystis* in Mugla.

The aim of the present study was to determine the subtypes of *Blastocystis* isolated from school-aged children in Mugla and to investigate the possible risk factors and symptoms related to *Blastocystis* infection.

## Materials and Methods

### Samples

Local Ethical Committee of Mugla Sitki Kocman University (17.10.2014) approved the present study. The written informed consents were received from all participants and their parents.

This cross-sectional study was conducted among primary school children in Mugla between Mar and Jun 2014. The schools were selected by simple random sampling. Mugla is located in southwest of Turkey, with a population of 866.665. Tourism and agriculture are the main sources of income. Mediterranean climate reigns in this region characterized by mild winters and hot, dry summers (14).

A single stool sample was collected from each student in clean, plastic screw-cap tubes without any fixatives. A questionnaire was developed for acquiring the demographic characteristics of patients’ factors which included: gender, age, residence, income, number of children and rooms at their home, homeownership, education of parents, occupations of mother, drinking water supply, and hygiene habits. Furthermore, we also investigate the following symptoms: lack of appetite, salivation during sleeping, headache, perianal itching, history of parasitic infections, teeth grinding, constipation, abdominal pain and nausea/vomiting in the questionnaire.

### Microscopy and cultivation of Blastocystis

Stool samples were examined by direct microscopy (DM) of native (0.9% Serum physiological) and Lugol’s iodine preparations upon arrival at the laboratory. Approximately 50 mg of fresh samples were inoculated into 3 ml of Jones medium supplemented with 10% fetal calf serum and incubated at 37 °C in standard bacteriological incubator. The cultures were checked by DM on third day and *Blastocystis* positive samples were subcultured in order to reduce faecal materials.

### PCR and sequencing

After third day of inoculation, subcultures were centrifuged at 12000 gr for one minute and genomic DNA was isolated with DNAzol kit (Thermo Fisher Scientific, Waltham, MA USA) from pellets according to the manufacturer’s instructions. The *Blastocystis* barcode region of SSU-rDNA gene was amplified in a single PCR with the primers RD5 and BhRDr (15) and with a gel documentation system (Infinity, Vilber Lourmat). The reaction was set in a 30-μl volume containing: 1–2 μl of template DNA, 0.4 pmol of each of the primers, 0.3 U of Taq DNA polymerase (Fermentas), 0.2 mM of each dNTP (Fermentas), 1× Taq buffer with (NH_4_)_2_SO_4_ (Fermentas). PCR amplifications were purified and sequenced by a commercial facility (MedSanTek, Istanbul) by using Applied Biosystems 377 DNA Sequencer.

### Identification of Blastocystis STs

The sequences were compared to *Blastocystis* SSU-rDNA sequences in GenBank nucleotide database using BLAST tool at the National Center for Biotechnology Information website (16). Additionally, the sequences were queried against the *Blastocystis* Sequence Typing web-site database (https://pubmlst.org/blastocystis/), developed by Keith Jolley and sited at the University of Oxford (17). The STs of isolates were determined according to the exact or closest matches (18).

Sequences were aligned with references by using ClustalW algorithm in Molecular Evolutionary Genetics Analysis version 6.0 (MEGA) and a phylogenetic tree was constructed using the Neighbor-Joining method in the bootstrap test (1000 replicates), the evolutionary distances were computed using the Maximum Composite Likelihood (19, 20). *Proteromonas lacertae* 18S ribosomal RNA gene partial sequence (AY224080) was used as out-group in phylogenetic analysis.

### Statistical analysis

SPSS (ver. 13.0 Chicago, IL, USA) was used for statistical analysis. The categorical variables were compared with Fisher’s exact and Pearson chi-square tests and significance level was set as two-sided *P*-value <0.05.

## Results

Overall, 468 students (ages varying from 6 to 11, mean 8.4±1.2) participated in the present study from four different schools in Mugla. The frequency of *Blastocystis* among children was 5.5% (26 out of 468) by DM and 7.4% (35 out of 468) by XIVC. The “barcode region” of *Blastocystis* SSU-rDNA gene was amplified in all of 35 culture positive samples and sequenced. The sequences were deposited to GenBank with accession numbers: KU361300-16.

**Fig. 1:**
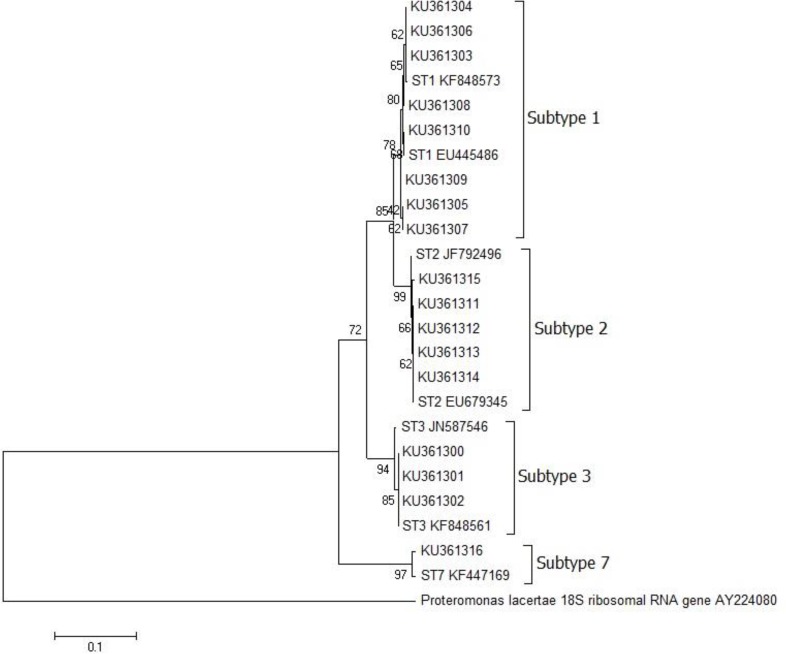
The phylogeny of *Blastocystis* isolates. KU361300-16 Genbank accession numbers of our isolates. Others represent references from Genbank with known subtype

After alignment and phylogenetic analysis of 35 sequences, the STs could be identified in 33 (94.2%) samples; 12 (34.2%) were ST3, 11 (31.4%) were ST1, 9 (25.7%) were ST2 and one (2.8%) ST7, remaining 2 (5.7%) sequences, nonspecific to *Blastocystis*, were eliminated. The phylogeny of *Blastocystis* isolates inferred from SSU rRNA gene sequences was shown in the Fig. 1. Finally, any of risk factors and symptoms was statistically related to *Blastocystis* infection (Table 1 and 2).

**Table 1:** The descriptive and statistical analysis of possible risk factors for *Blastocystis* infection

***Variables***		***No. of examined***	***No. of infected***	***(%)***	***χ^2^***	***P-value***
Gender	Male	222	17	7.7	0.20	1.000
	Female	246	18	7.3		
Age (yr)	7	130	13	10.0		
	8	106	4	3.8	3.324	0.344
	9	116	9	7.8		
	10	116	9	7.8		
Residence	Urban	297	18	6.1	2.362	0.124
	Rural	171	17	9.9		
No. of children at home	1	103	7	6.8	1.131	0.568
	2	288	20	6.9		
	≥ 3	77	8	10.4		
No. of rooms at home	2	52	3	4.8		
	3	178	12	6.7	0.653	0.721
	≥4	238	20	8.4		
Home ownership	Owner	282	26	9.2	3.109	0.54
	Renter	186	9	4.8		
Drinking water supply	Tap/Bottled	428	29	6.8	3.576	0.059
	Well/spring	40	6	15		
	Low	219	16	7.3	2.614	0.271
Income*	Moderate	154	15	9.7		
	High	95	4	4.2		
Occupation of mother	Worker/civil servant	134	8	6.0	0.617	0.432
	Housewife	334	27	8.1		
The education of mother	Literate/Primary	262	20	7.6		
	Secondary school	53	5	9.4		
	High school	110	8	7.3	0.806	0.848
	University	43	2	4.7		
The education of father	Literate/Primary	221	19	8.6		
	Secondary school	58	5	8.6	2.69	0.441
	High school	122	5	4.1		
	University	67	6	9.0		
Hand washing	Soap	432	31	7.2	0.744	0.388
	Only water	36	4	11.1		

* Monthly income of families; low: 0–250$, modarate: 251–500$, high: ≥500$

**Table 2:** The descriptive and statistical analyses of symptoms

	***Infected group*** *(n=35)*		***Non-infected group*** *(n=433)*			
Symptoms	**No.**	**(%)**	**No.**	**(%)**	**χ^2^**	***P*-value**
Lack of appetite	9	25.7	121	27.9	0.080	0.777
Salivation during sleeping	12	34.3	130	30	0.278	0.598
Headache	5	14.3	58	13.4	0.022	0.882
Perianal itching	5	14.3	102	23.6	1.578	0.209
History of parasitic infections	4	11.4	23	5.3	2.229	0.135
Teeth grinding	3	8.6	86	19.9	2.680	0.102
Constipation	6	17.1	61	14.1	0.246	0.620
Abdominal pain	6	17.1	129	29.8	2.524	0.112
Nausea/vomiting	1	2.9	12	2.8	0.001	1.00

## Discussion

Since children are most vulnerable group to acquire parasitic infections, they are primarily the main targets of control programs and epidemiological studies (21). In the present study, 7.4% of students were infected with *Blastocystis*. The frequency of *Blastocystis* in child age groups ranged from 1.4% to 23.6% according to recent publications in Turkey (22, 23). However, most of these studies relied on the findings with DM of stool samples and suffered from the fact that it was the least sensitive method for diagnosis of *Blastocystis* in comparison with XIVC, conventional and real-time PCR methods (24–26).

In the present study, the positive rate of *Blastocystis* increased from 5.5% to 7.4% when XIVC was used. Thus, our study also highlighted the insensitivity of DM as compared to XIVC. A limitation of our study was that we extracted DNA from positive cultures and did not perform PCR assay directly from stool samples. Thus, the positive rate of *Blastocystis* might be higher than we detected because there might be some slow-growing *Blastocystis* that caused overgrowth of one subtype of another in mixed infections (27). PCR-based detection directly from stool was more sensitive than XIVC (28). A similar result was also reported by Stensvold et al. (29).

A real-time PCR assay was reported by far the most sensitive and allowed to ST determination by direct sequencing of qPCR products (9). DM and XIC were showed only 29% and 52% sensitivity, respectively.

In recent years, there has been a growing interest in *Blastocystis* all around the world, primarily after the identification of STs. Although many studies carried out about the STs in adult age groups, few studies have attempted to investigate *Blastocystis* STs in children (1). We identified four different subtypes of *Blastocystis* (ST1, ST2, ST3, and ST7) in children and ST3 was responsible for the great majority of infections. All children in a study were infected with *Blastocystis* and ST3 was the most abundant genotype followed by ST1, ST2, and ST4 (30). In another study, ST1 was found as the most frequent *Blastocystis* genotype in Nepali children (31). In Turkey, the frequency of *Blastocystis* was 31.7% among children and 32.8% among adults, the difference was not statistically significant. In this study, ST3 was the most common ST in both of the groups (32). *Blastocystis* STs were investigated in different cities of Turkey by STS PCR, sequencing and real-time PCR in different populations including IBS patients, symptomatic and asymptomatic people ST1, ST 2, ST 3, ST 4, ST 6, and ST 7 were identified (12, 13, 33). ST3 was found the predominant genotype in a variety of populations as in the present study (12, 34, 35). In general, despite the variety of study populations, our results were consistent with the other studies in Turkey and other countries.

In the present study, none of the investigated parameters was found to be associated with *Blastocystis* infection. The factors that influence *Blastocystis* frequency have been investigated and many factors reported: age, nutritional status, crowded life area, close contact with animals, poor hygiene practices, poverty, consumption of contaminated food or water (8, 27, 36). We found no relationship between gastrointestinal symptoms and the presence of *Blastocystis*. In a number of studies, *Blastocystis* was linked to nonspecific symptoms including abdominal pain, diarrhea, nausea vomiting, and flatulence (27, 37). It was not possible to investigate subtype related pathogenicity due to the small size of positive cases (35 children).

## Conclusion

Our finding on *Blastocystis* STs in children, the predominance of ST3, was similar to those reported in other studies despite the variety of study populations. Moreover, our findings of symptoms and risk factors also increased the questions about *Blastocystis*. Therefore, further research dealing with these issues would be worthwhile.

## References

[1] Wawrzyniak I Poirier P Viscogliosi E *Blastocystis*, an unrecognized parasite: an overview of pathogenesis and diagnosis. Ther Adv Infect Dis. 2013; 1(5):167–78. 25165551 10.1177/2049936113504754 PMC4040727

[2] El Safadi D Meloni D Poirier P Molecular epidemiology of *Blastocystis* in Lebanon and correlation between subtype 1 and gastrointestinal symptoms. Am J Trop Med Hyg. 2013; 88(6):1203–6. 23458955 10.4269/ajtmh.12-0777 PMC3752823

[3] Coyle CM Varughese J Weiss LM *Blastocystis*: to treat or not to treat. Clin Infect Dis. 2012; 54(1):105–10. 22075794 10.1093/cid/cir810

[4] Scanlan PD Stensvold CR *Blastocystis*: getting to grips with our guileful guest. Trends Parasitol. 2013;29(11):523–9. 24080063 10.1016/j.pt.2013.08.006

[5] Jimenez-Gonzalez DE Martinez-Flores WA Reyes-Gordillo J *Blastocystis* infection is associated with irritable bowel syndrome in a Mexican patient population. Parasitol Res. 2012;110(3):1269–75. 21870243 10.1007/s00436-011-2626-7

[6] Zuel-Fakkar NM Abdel Hameed DM Hassanin OM Study of *Blastocystis* hominis isolates in urticaria: a case-control study. Clin Exp Dermatol. 2011;36(8):908–10. 21790724 10.1111/j.1365-2230.2011.04127.x

[7] Tan TC Ong SC Suresh KG Genetic variability of *Blastocystis* sp. isolates obtained from cancer and HIV/AIDS patients. Parasitol Res. 2009;105(5):1283–6. 19603182 10.1007/s00436-009-1551-5

[8] Stensvold CR Lewis HC Hammerum AM *Blastocystis*: unravelling potential risk factors and clinical significance of a common but neglected parasite. Epidemiol Infect. 2009; 137(11):1655–63. 19393117 10.1017/S0950268809002672

[9] Poirier P Wawrzyniak I Albert A Development and evaluation of a real-time PCR assay for detection and quantification of *Blastocystis* parasites in human stool samples: prospective study of patients with hematological malignancies. J Clin Microbiol. 2011;49(3):975–83. 21177897 10.1128/JCM.01392-10 PMC3067686

[10] Alfellani MA Taner-Mulla D Jacob AS Genetic diversity of *Blastocystis* in livestock and zoo animals. Protist. 2013;164(4):497–509. 23770574 10.1016/j.protis.2013.05.003

[11] Stensvold CR Comparison of sequencing (barcode region) and sequence-tagged-site PCR for *Blastocystis* subtyping. J Clin Microbiol. 2013; 51(1):190–4. 23115257 10.1128/JCM.02541-12 PMC3536234

[12] Dagci H Kurt Ö Demirel M Epidemiological and Diagnostic Features of *Blastocystis* Infection in Symptomatic Patients in Izmir Province, Turkey. Iran J Parasitol. 2014;9(4):519–29. 25759733 PMC4345091

[13] Ozyurt M Kurt O Mølbak K Molecular epidemiology of *Blastocystis* infections in Turkey. Parasitol Int. 2008;57(3):300–6. 18337161 10.1016/j.parint.2008.01.004

[14] Turkish Statistical Institute Report no. 4200. Secilmis gostergelerle Mugla 2013. TUIK: Ankara, 2014.

[15] Scicluna SM Tawari B Clark CG DNA barcoding of *Blastocystis*. Protist. 2006;157(1):77–85. 16431158 10.1016/j.protis.2005.12.001

[16] Altschul SF Gish W Miller W Basic local alignment search tool. J Mol Biol. 1990;215(3):403–10. 2231712 10.1016/S0022-2836(05)80360-2

[17] Jolley KA Maiden MC BIGSdb: Scalable analysis of bacterial genome variation at the population level. BMC Bioinformatics. 2010;11:595. 21143983 10.1186/1471-2105-11-595 PMC3004885

[18] Stensvold CR Suresh GK Tan KS Terminology for *Blastocystis* subtypes-a consensus. Trends Parasitol. 2007;23(3):93–6. 17241816 10.1016/j.pt.2007.01.004

[19] Saitou N Nei M The neighbor-joining method: a new method for reconstructing phylogenetic trees. Mol Biol Evol. 1987;4(4):406–25. 3447015 10.1093/oxfordjournals.molbev.a040454

[20] Tamura K Stecher G Peterson D MEGA6: Molecular Evolutionary Genetics Analysis version 6.0. Mol Biol Evol. 2013;30(12):2725–9. 24132122 10.1093/molbev/mst197 PMC3840312

[21] Harhay MO Horton J Olliaro PL Epidemiology and control of human gastrointestinal parasites in children. Expert Rev Anti Infect Ther. 2010;8(2):219–34. 20109051 10.1586/eri.09.119 PMC2851163

[22] Celik T Daldal N Karaman U Incidence of intestinal parasites among primary school children in Malatya. Turkiye Parazitol Derg. 2006; 30(1):35–8. 17106853

[23] Göz Y Aydin A Tuncer O Distribution of intestinal parasites in children from the 23 Nisan Primary School in Hakkari. Turkiye Parazitol Derg. 2005;29(4):268–70. 17124686

[24] Moosavi A Haghighi A Mojarad EN Genetic variability of *Blastocystis* sp. isolated from symptomatic and asymptomatic individuals in Iran. Parasitol Res. 2012;111(6):2311–5. 22948205 10.1007/s00436-012-3085-5

[25] Roberts T Barratt J Harkness J Comparison of microscopy, culture, and conventional polymerase chain reaction for detection of *Blastocystis* sp. in clinical stool samples. Am J Trop Med Hyg. 2011;84(2):308–12. 21292905 10.4269/ajtmh.2011.10-0447 PMC3029188

[26] Suresh K Smith H Comparison of methods for detecting *Blastocystis hominis*. Eur J Clin Microbiol Infect Dis. 2004;23(6):509–11. 15168139 10.1007/s10096-004-1123-7

[27] Tan KS New insights on classification, identification, and clinical relevance of *Blastocystis* spp. Clin Microbiol Rev. 2008;21(4):639–65. 18854485 10.1128/CMR.00022-08 PMC2570156

[28] Parkar U Traub RJ Kumar S Direct characterization of *Blastocystis* from faeces by PCR and evidence of zoonotic potential. Parasitology. 2007;134(Pt 3):359–67. 17052374 10.1017/S0031182006001582

[29] Stensvold R Brillowska-Dabrowska A Nielsen HV Detection of *Blastocystis hominis* in unpreserved stool specimens by using polymerase chain reaction. J Parasitol. 2006;92(5):1081–7. 17152954 10.1645/GE-840R.1

[30] El Safadi D Gaayeb L Meloni D Children of Senegal River Basin show the highest prevalence of *Blastocystis* sp. ever observed worldwide. BMC Infect Dis. 2014;14:164. 24666632 10.1186/1471-2334-14-164 PMC3987649

[31] Yoshikawa H Wu Z Pandey K Molecular characterization of *Blastocystis* isolates from children and rhesus monkeys in Kathmandu, Nepal. Vet Parasitol. 2009;160(3–4):295–300. 19136214 10.1016/j.vetpar.2008.11.029

[32] Dogruman-Al F Dagci H Yoshikawa H A possible link between subtype 2 and asymptomatic infections of *Blastocystis hominis*. Parasitol Res. 2008;103(3):685–9. 18523804 10.1007/s00436-008-1031-3

[33] Dogruman-Al F Yoshikawa H Kustimur S PCR-based subtyping of *Blastocystis* isolates from symptomatic and asymptomatic individuals in a major hospital in Ankara, Turkey. Parasitol Res. 2009;106(1):263–8. 19847459 10.1007/s00436-009-1658-8

[34] Stensvold CR *Blastocystis*: Genetic diversity and molecular methods for diagnosis and epidemiology. Trop Parasitol. 2013;3(1):26–34. 23961438 10.4103/2229-5070.113896 PMC3745667

[35] Ertuğ S Malatyal E Ertabaklar H Sub-type distribution of *Blastocystis* isolates and evaluation of clinical symptoms detected in Aydin province, Turkey. Mikrobiyol Bul. 2015;49(1):98–104. 25706735 10.5578/mb.8532

[36] Pipatsatitpong D Rangsin R Leelayoova S Incidence and risk factors of *Blastocystis* infection in an orphanage in Bangkok, Thailand. Parasit Vectors. 2012;5:37. 22330427 10.1186/1756-3305-5-37 PMC3299613

[37] Dinleyici EC Eren M Dogan N Clinical efficacy of Saccharomyces boulardii or metro-nidazole in symptomatic children with *Blastocystis hominis* infection. Parasitol Res. 2011;108(3):541–5. 20922415 10.1007/s00436-010-2095-4

